# Rayleigh Wave Calibration of Acoustic Emission Sensors and Ultrasonic Transducers

**DOI:** 10.3390/s19143129

**Published:** 2019-07-16

**Authors:** Kanji Ono

**Affiliations:** Department of Materials Science and Engineering, University of California, Los Angeles (UCLA), Los Angeles, CA 90095, USA; ono@ucla.edu; Tel.: +1-310-825-5534

**Keywords:** acoustic emission sensors, ultrasonic transducers, calibration, receiving sensitivities, Rayleigh waves, normally incident waves, guided waves, FEM calculation

## Abstract

Acoustic emission (AE) sensors and ultrasonic transducers were characterized for the detection of Rayleigh waves (RW). Small aperture reference sensors were characterized first using the fracture of glass capillary tubes in combination with a theoretical displacement calculation, which utilized finite element method (FEM) and was verified by laser interferometer. For the calibration of 18 commercial sensors and two piezoceramic disks, a 90° angle beam transducer was used to generate RW pulses on an aluminum transfer block. By a substitution method, RW receiving sensitivity of a sensor under test was determined over the range of frequency from 22 kHz to 2 MHz. Results were compared to the sensitivities to normally incident waves (NW) and to other guided waves (GW). It was found that (1) NW sensitivities are always higher than RW sensitivities, (2) differences between NW and RW receiving sensitivities are dependent on frequency and sensor size, (3) most sensors show comparable RW and GW receiving sensitivities, especially those of commonly used AE sensors, and (4) the receiving sensitivities of small aperture (1 mm diameter) sensors behave differently from larger sensors.

## 1. Introduction

Acoustic emission (AE) technology plays a key role in structural health monitoring (SHM), as discussed in several reviews [1,2,3,4,5,6,7,8]. In AE monitoring and ultrasonic testing (UT) various types of waves are utilized. Most common is longitudinal waves, followed by transverse (or shear) waves and various guided waves. In both AE and UT inspection of large structures, such as concrete dams and bridges and heavy wall pressure vessels, longitudinal waves play the central role for detecting volumetric flaws. New approaches with embedded and microelectromechanical sensors are also utilized in standard techniques [9,10,11]. For thin structural elements, Lamb (or plate) waves are important, while Rayleigh (or surface) waves provide the means to interrogate medium to heavy walled structures for locating and evaluating near-surface defects. Recent developments [12,13,14,15] using guided waves for various inspection goals provided expanded avenues for deployment. For basic test methods with different wave modes, see standard textbooks for UT and nondestructive evaluation and references [1,2,3]. In using AE techniques, AE sensors are essential components in detecting low level elastic vibrations of damages occurring in structural elements. Because of their importance, many studies on sensor characteristics have been conducted and reported [16,17,18,19,20,21,22,23,24,25].

**Figure 1:**
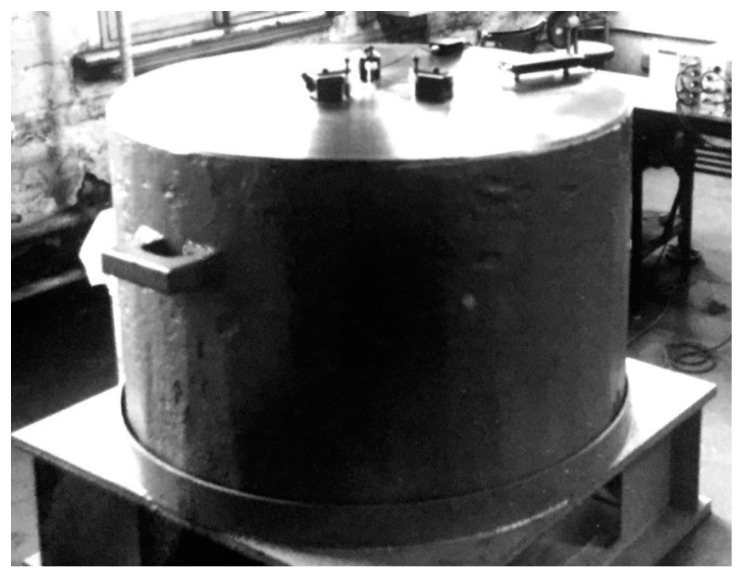
Steel block (1.1 m diameter, 0.76 m thickness) used for AE sensor calibration. Photograph provided by T. Watanabe of Nippon Steel Corp., Tokyo, Japan.

**Figure 2:**
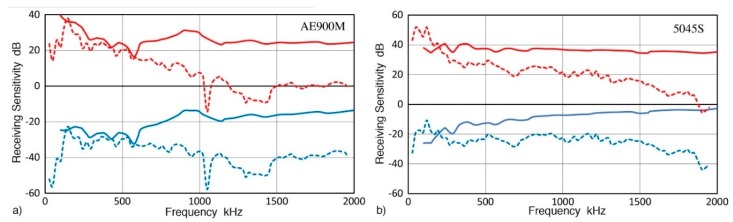
(**a**) Laser interferometric calibration results of Matsuda et al. [34,35]. NW (solid curve) and RW (dash curve) receiving sensitivities for AE900M sensor. Upper red curves are for the velocity sensitivity and the lower blue curves are for the displacement sensitivity. (**b**) 5045S sensor. Sensor types were identified with the assistance of the lead author, Y. Matsuda.

A series of recent AE sensor works clarified their characteristics when the wave motions to be detected are from normally incident longitudinal waves (NW) or from guided waves travelling on plates or bars [26,27,28,29,30]. These works relied on laser interferometry as the basis of displacement calibration, as prescribed for vibration sensors in ISO16063-11, but using pulse excitation [31]. Numerical data of the receiving sensitivities to NW have recently been made available for over 40 sensors [32]. The method of determining the receiving sensitivities to Rayleigh (or surface) waves (RW) was standardized in the 1980s on the basis of extensive investigation at the National Institute of Standards and Technology (NIST) [16,17,18,19,24,25,33]. This method used the fracture of glass capillary tubes as the signal source and a capacitive displacement sensor as the standard receiver. The calibration of the standard receiver relied on elasticity theory using the fracture force as an input parameter. It requires a large steel block as the wave propagation medium (0.9 m diameter and 0.4 m thickness for the NIST studies). To date, only a few blocks have been used for sensor calibration studies. Figure 1 shows one such block at Nippon Steel Corp. (Tokyo, Japan), the size of which was 1.1 m diameter and 0.76 m thickness. Actually, wave reflections limited signal duration to less than 100 µs even using these large blocks [17]. At this time, however, this calibration method [24] is supported by no national standards agency, and secondary calibration [25] can only be conducted using primary calibration of more than ten years old. Another study by Matsuda et al. [34,35] used laser interferometry directly on an aluminum transfer block, receiving Rayleigh waves generated by a line-focused Q-switched YAG laser. This reference source provided ±1 dB flatness over 0.06 to 3 MHz and eight sensors were characterized. However, sensor types remain unknown except for three, which also were evaluated for NW sensitivities [34,35]. Presently, these three are the only commercial AE sensors that have been calibrated for both NW and RW receiving sensitivities with laser references. Figure 2a shows the NW and RW receiving sensitivities for one of them, AE900M, made by Fuji Ceramics (Fujinomiya, Japan). The data were read from published figures and plotted for both displacement and velocity sensitivities. The NW (solid curves) and RW (dash curves) sensitivities are close below 0.6 MHz, but diverged as frequency increased. NW and RW receiving sensitivities for another broadband sensor (5045S, Fuji Ceramics, Fujinomiya, Japan), shown in Figure 2b, behaved differently, hardly matching between the two wave modes. It appears that this direct laser method has not been reproduced elsewhere for the case of RW sensitivity calibration even though laser interferometers have become more widely available today. Still, these are expensive and uncommon in AE laboratories. Thus, it is necessary to seek additional methods to obtain the receiving sensitivities to Rayleigh waves and explore the differences between the receiving sensitivities to NW and RW for various AE sensors. 

Another approach, called reciprocity methods, has been used by a group of AE workers [36,37,38,39,40]. This is based on classical acoustic reciprocity calibration methods, which are applicable to electrodynamic and electrostatic speakers and microphones [41]. A typical implementation uses three identical reversible transducers. It is assumed that their transmission and receiving sensitivities, S_i_ and M_i_ (i = 1 to 3), respectively, are reciprocal. Here, S_i_ is defined for acoustic pressure at the receiver position per unit input. This makes the ratio of S_i_ and M_i_, called reciprocity parameter, H = S_i_/M_i_, to be equal to the transfer function of the wave propagation medium [36,42,43]. When the reciprocity method is applied to a solid medium, H has been taken as the Green function defined for a point source and a point receiver [36]. With disk shaped transducers for the case of longitudinal waves, the inverse of H corresponds to the Lommel integral, which strongly depends on the disk area, wavelength and propagation distance [44]. This integral is also known as diffraction correction integral and its values for typical AE sensor size (12.7 mm) and propagation distance (250 mm) differ from 1/H by 60.5 dB. Thus, the use of the Green function causes unacceptable error since the sensitivities are proportional to 1/√H. There is another issue in applying the reciprocity methods to AE sensors. For AE sensors and ultrasonic transducers, transmission sensitivities are defined on their face as T_i_. Transmitter output in displacement or velocity can be measured directly by laser interferometry. For all ultrasonic transducers and AE sensors tested, T_i_ was found to differ from M_i_ [27], rendering the definition of H into question. As S_i_ = H × T_i_ with proper unit conversion, H makes sense only when T_i_ = M_i_. In most of the reciprocity studies [36,37,38,39,40]; however, these three issues have been ignored, making their results invalid. They also violated the basic requirement of sensor size being much smaller than the wavelength [41]. Hill and Adams [42] showed that reciprocity methods can be modified even when T_i_ and M_i_ are unequal, but one needs to know the ratio of T/M for one of the three transducers. This Hill–Adams method was recently verified to provide identical calibration as the laser-based calibration methods discussed above [27]. For the case of RW calibration, corresponding diffraction loss analysis is unavailable and aperture effects further complicate meaningful calibration. That is, reciprocity methods are inapplicable for RW calibration at present.

The third approach involves the modeling of wave generation and detection, in combination with laser interferometry. The modeling for guided wave propagation often utilizes finite element methods (FEM) and semi-analytical finite element (SAFE) method [45]. Using FEM, Hamstad and coworkers have examined guided wave generation and propagation from pencil lead breaks with µs-order rise times [46,47,48,49]. They considered monopole and dipole point step forces and obtained waveforms and frequency spectral information on thin and thick plates. Sause extended this FEM approach to anisotropic fiber composite plates. For sensor calibration, Sause and Hamstad [23] obtained normal displacements from such a step force for NW, RW, Lamb waves, and rod waves. For a small sensor (with 1 mm diameter sensing area), they found identical sensor sensitivities for all the wave types. For this work, they utilized laser interferometry for displacement amplitude determination and verified the results of FEM calculations. 

Using the verified displacement calculations with FEM of Sause and Hamstad [23], the normal displacement from Rayleigh wave on a large transfer block can be predicted when the magnitude of step force applied at the origin can be determined. While constructing a mechanical loading mechanism and measuring force changes during a fast (~1 µs) fracture event require elaborate and costly efforts, a much simpler design for breaking glass capillary tubes can be devised. This loading device can be built in a typical mechanical laboratory as will be shown in the following section. The core concept of this device is based on the nature of glass fracture. It is well known that common sodium silicate glass suffers from static fatigue and fractures after a certain time period upon the application of a tensile load [50]. This is also called delayed fracture and enables fracture force measurements without electronic instrumentation. 

This study first examines glass capillary fractures describing experimental set-up and results of force–time traces obtained by ultrasonic transducers. Next, small aperture AE sensors are characterized for their RW receiving sensitivities using measured fracture force and a theoretical displacement function from FEM calculation kindly supplied by M. Sause of University of Augsburg. This is followed by the use of a 90° angle beam ultrasonic transducer as the signal source, which produces directional RW pulses. This directivity allows the use of smaller transfer blocks in comparison to the omnidirectional sources, such as glass capillary fracture and common disk sensors. These two experimental refinements allow one to conduct verifiable Rayleigh wave calibration of AE and ultrasonic sensors outside the NIST and other national laboratories for the first time. With this RW source, 18 additional types of AE sensors and ultrasonic transducers are tested for their RW receiving sensitivities. The receiving sensitivities to RW, NW and other guided waves (GW) of the 18 sensors are compared. Such comparison has not been reported previously. These sections are followed by further discussion and conclusions. 

## 2. Glass Capillary Fracture

The time history of force due to fracture of glass capillary (GC) tube and other sources was first studied in detail at NIST [19,20]. They determined the source functions through the deconvolution of measured displacement. This NIST study reported the rise time as low as 0.20 µs for a GC tube of 0.20 mm diameter, but the current ASTM standard E1106 [24] indicates the rise time of 0.2 to 0.3 µs for GC tubes of 0.2 ± 0.1 mm diameter to be the best estimate. The duration of GC fracture is governed by the terminal crack velocity of sodium silicate glass, which was found to be 1.51 mm/µs [51]. When crack velocity is assumed to linearly increase under steady stress after the initiation of a crack, the average crack velocity is one half of the terminal velocity. This gives 0.4 µs for a 0.2 mm diameter GC tube when a crack initiates at a contact point. Burks’ FEM analysis of GC fracture favors inner sidewalls of a GC tube as the highest tensile stress and likely crack initiation points [52]. If so, a crack is likely to start at the mid-point, shortening the time by a half or 0.2 µs for 0.2 mm diameter GC tube. Using a pair of 5-MHz ultrasonic transducers, the transit time through a GC tube of 0.36 mm diameter was measured to be 0.12 µs. With Gilman’s value of 1.51 mm/µs, linear velocity change assumption and the longitudinal wave velocity for sodium silicate glass of 5.43 mm/µs [53], it takes a crack 0.62 µs to cross the GC tube, assuming that the fracture force remains on the tube. More recent value of the terminal crack velocity is 1.43 mm/µs [53], which adds 5% to the rise time. This can be considered an estimate of GC fracture rise time for 0.36 mm GC tubes. This value is twice that of the rise time of 0.3 µs used in the FEM calculation [23]. This reflects a diameter increase by 1.8 times in this ultrasonic measurement. Thus, the two rise time values are in agreements.

Gary and Hamstad [46] analyzed the elastic displacements on a plate resulting from GC and pencil lead fracture by first representing the source function, initially with a linear function and later by a cosine bell function [47,51]. This function is given as

F(t) = 0.5 F_m_[1 − cos(πt/t_r_)] for t ≤ t_r_   = F_m_        for t > t_r_ (1)

where F is force, F_m_ is maximum force, t is time, and t_r_ is rise time, respectively. This has been used to represent the fracture of GC tubes in the FEM studies by Hamstad and coworkers [23,47,48,49].

The fracture of glass capillary tubes is examined here as a source of fast step force at a point and of resultant Rayleigh waves on a transfer block. Glass capillary tubes used in this study were supplied by Shanghai Great Wall Instrument Co., Shanghai, China. Measured outside diameters typically ranged from 0.32 to 0.40 mm. GC tubes were selected and actual diameter values used were 0.36 ± 0.02 mm. The wall thickness was approximately 0.05 mm, while the length was 100 mm.

**Figure 3:**
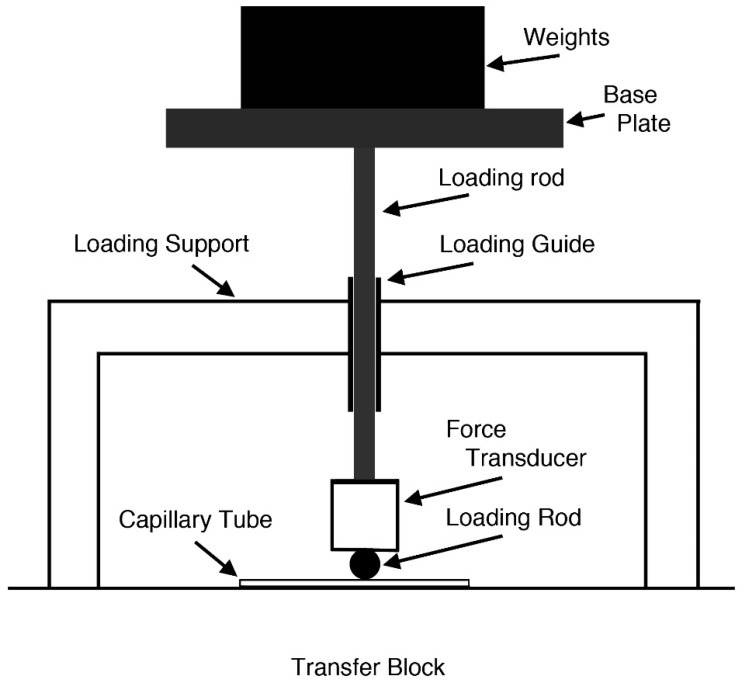
Schematic drawing of a loading device.

**Figure 4:**
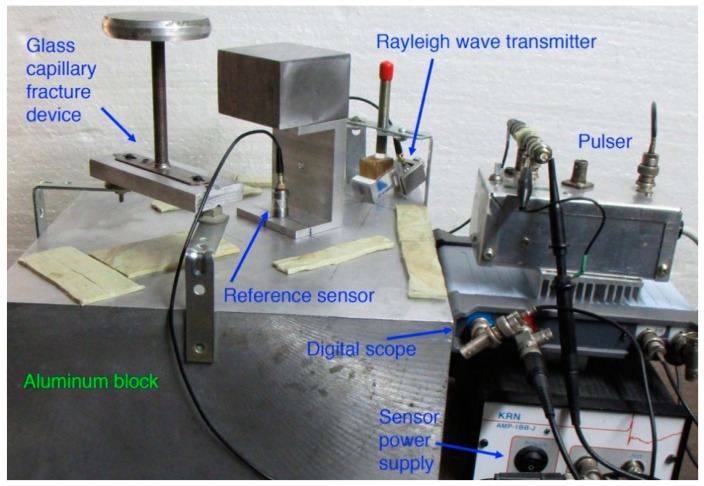
Photograph of Rayleigh wave calibration set-up (center) and a part of GC tube fracture set-up (left side). Rayleigh wave transmitter is placed at the back corner of the block and a reference sensor (under a steel block) is at 100 mm from the transmitter. Also shown are a pulser, a digital oscilloscope, a sensor power supply, and vibration damping strips.

Loading devices were used to apply static force using a combination of metal weights, which ranged from 20 g to 600 g. For the GC tubes used, 10–20 N fracture force was required and typically 10–15 weights of various values were utilized, in 20 g step nearing fracture. A schematic drawing of the loading device is given in Figure 3. Loading rod that contacts with a GC tube was 1.99 mm diameter drill rod. This rod had a ground flat glued to a force transducer (Olympus V112 ultrasonic transducer, 10 MHz center frequency, 6.4 mm diameter; Olympus NDT, Waltham, MA, USA). The output of V112 transducer was used to monitor irregularities in force–time curves. Upon fracture, the total weight of loading elements, including base plate, loading rod, force transducer, connecting rod and weights, is used as the fracture force. The total weight needs to be close to the eventual fracture load within a few % for the waiting time to be relatively short (5 to 30 s). Nearing anticipated fracture, load increment of about 20 g was used by switching weights of various values. When static fatigue produces a delayed fracture of a GC tube after at least a few seconds of sustained loading following the last weight increment, the fracture force is obtained from the total weight. When a GC fractured immediately upon a load increment, this test was discarded as fracture force cannot be determined. Figure 4 is a photograph of Rayleigh wave calibration set-up (center) and a part of GC tube fracture set-up (left side). On the left side of a large aluminum block, loading support, loading guide and base plate for a GC tube loading device are visible.

**Figure 5:**
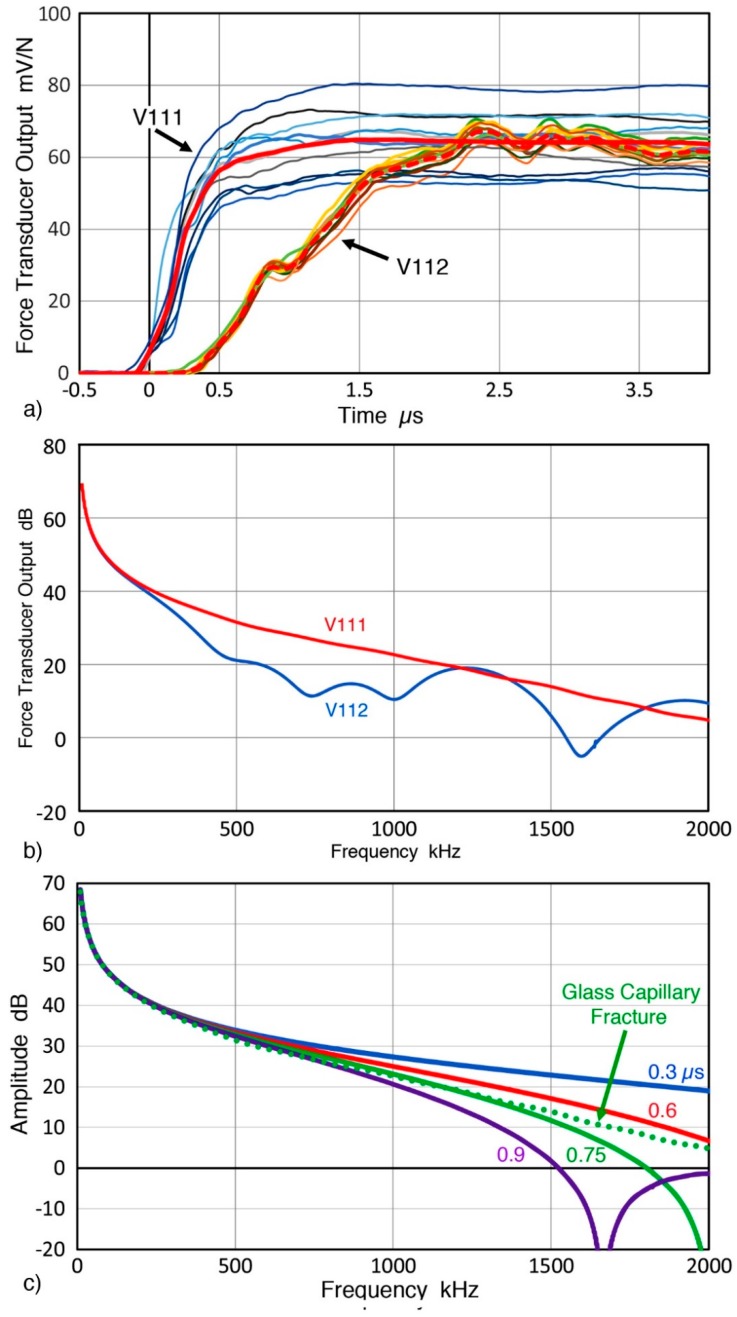
(**a**) The output voltages from force transducers (V111 and V112) vs. time during the fracture of 10 GC tubes. (**b**) Fast Fourier-transform (FFT) spectra of the force–time curves in Figure 5a. (**c**) FFT of V111 in Figure 5b is given in green dot curve and FFT of cosine bell functions of four rise times in solid curves. Rise time values are 0.3 µs (blue), 0.6 µs (red), 0.75 µs (green), and 0.9 µs (purple), respectively.

When a GC tube is fractured on the sensing face of an ultrasonic transducer, the transducer output appears to represent a force–time curve of the GC tube fracture, as shown in Figure 5a. Here, Olympus V111 transducer (10-MHz, 12.7 mm diameter) was used as another force transducer together with V112, which is a part of the loading device. As noted above, the loading rod is glued to V112 and causes a delay of 0.33 µs. The outputs from the transducers were normalized by the fracture force. Ten curves are plotted with their average (red curve for V111 and red dash curve for V112). The V111 output showed fast rise, followed by a plateau after 1.5 µs. The averaged curve indicated a rise time of approximately 0.5 µs from the start to a sharp bend, followed by slow rise lasting ~1 µs. The V112 output is delayed by ~0.4 µs, corresponding to the loading rod diameter of 2 mm plus a delay of 0.05 to 0.1 µs, with a rise time of 0.5 µs to the first small peak. This is followed by slow rise to a plateau at about 2.3 µs. The rise time of GC fracture is at least 0.5 µs from the two force–time curves. The delay time is longer than the ultrasonic transit time measured (0.33 µs through the 2 mm steel loading rod) and this difference indicates that the GC fracture did not start from the contact point of the GC tube and the loading rod. In fact, the extra delay implies that the fracture initiation point is slightly below the mid-point, predicted by the Burks calculation [52]. The plateau values of the two averaged curves are comparable at approximately 65 mV/N. While the output plateau for V111 varied by ±25%, V112 output was more consistent and the deviation from the average was less than ±10%. While additional work is needed to verify if the V111 output gives the force–time curve of GC fracture, the V112 output can be used to monitor the condition of fracture since it is a part of the loading mechanism. The use of 10-MHz transducers is expected to follow fast fracture with the time resolution down to 0.05 µs.

The fast Fourier-transform (FFT) results of the two averaged force–time curves are shown in Figure 5b. The overall shape comes from the stepwise increase and the spectrum for V111 transducer decreased smoothly with increasing frequency. Reflecting the presence of a steel rod between the GC tube and the sensing face of V112 transducer, its spectrum deviated from that of V111 with four dips. The V111 spectrum was compared to corresponding spectra of cosine bell force–time curves with the rise times of 0.3, 0.6, 0.75, and 0.9 µs. Results for V111 output voltage (green dot) and cosine bell functions (solid curves with t_r_ values indicated in the figure) are given in Figure 5c. The maximum value of the cosine bell function was set equal to the value for GC fracture, F_m_ = 65 mV/N. In this case, F represents voltage output from V111 transducer, normalized with applied fracture force. The spectrum for GC fracture matched best with that of cosine bell with t_r_ = 0.75 µs. In this case, the cosine bell curve exhibits a dip at 2.003 MHz (only the initial part of the dip is shown in Figure 5c) and the two curves started to deviate for frequency, f > 1.35 MHz. This dip appears in all the cosine bell functions and can be seen at 1.667 MHz for the case of 0.9 µs rise time (purple curve). When the trend below 1.35 MHz is extrapolated, the GC fracture curve is represented well, with a good match to the 0.75 µs cosine bell. These dips come from the first null of an effective rectangular window function of length 2t_r_/3 (expressed by a sinc function) as the frequency of the dip corresponds to 1.500/t_r_. This justifies the spectral smoothing through extrapolation over the frequency range of the dip.

The observed agreement of FFT spectra of GC fracture and the cosine bell function suggests that the rise time value of 0.75 µs is the appropriate rise time, rather than 0.5 µs value from Figure 5a, which can be due to an intermediate step in fracture. This also is close to the rise time of 0.62 µs predicted from the longitudinal wave and crack velocities in glass. For sensor calibration, rise time effects between 0.62 and 0.75 µs are less than 2 dB below 1.5 MHz, but this issue must be examined further using other experimental methods, including laser interferometry, in order to improve the basis of sensor calibration.

**Figure 6:**
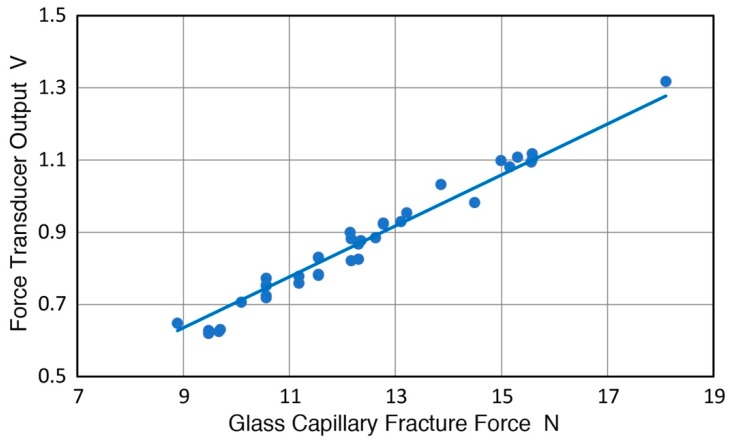
The output voltage from a force transducer (V112) vs. fracture force of GC tubes of 0.36 mm diameter.

During GC tube fracture tests for AE sensor calibration, only the V112 output is available. The maximum output data of 40 tests were plotted against the fracture force obtained from total applied weight. This is shown in Figure 6. The maximum output voltage is proportional to fracture force with the slope of 70.6 mV/N. The maximum output voltage is slightly (8.6%) higher than averaged plateau voltage, but it can be used as back-up for applied weight.

The fracture force measured for GC tubes used in the above tests averaged 12.30 N with the standard deviation of 2.15 N. This strength value is within the range specified in ASTM E1106 [24]. Using the simple estimation method of Weibull modulus, m, m is found to be 6.31 [54]. This agrees well with m of 5.74 reported for a sodium silicate glass [54]. The m value for GC tubes is 1/3 to ½ of those reported for pencil lead, reported by Higo and Inaba [55].

This evaluation of commercially supplied GC tubes found their fracture properties to be similar to the GC tubes used in earlier studies. However, the rise time of GC fracture is estimated to be 0.62 to 0.75 µs, higher than the previously reported values of 0.2 to 0.3 µs. This reflects the use of 1.8 times larger diameter GC tubes as noted above.

## 3. Rayleigh Wave Calibration of Reference Sensors

The basic approach of RW calibration in this work follows ASTM E1106 [24] with modifications. In ASTM E1106, RW signals are generated on a transfer block and the signals are measured with a reference transducer, simultaneously measuring the output from a sensor under test placed at an equivalent position. The first need is the calibration of a reference sensor. In this work, small aperture AE sensors with 1 mm diameter sensing area were calibrated. One was model KRNBB-PC with an integral amplifier and used with a power supply (AMP-1BB-J). The other was model KRNBB-PCP, directly connected to an oscilloscope input with 1 MΩ input impedance. These were supplied by KRN Services, Richland, WA, USA. One of them is placed on a transfer block of an aluminum alloy, using Vaseline as a couplant and with 30 N force, as shown in Figure 4. The transfer block was previously used in another sensor study [56]. Its size is 305 × 305 × 156 mm^3^ and surfaces were ground and polished. At 100 mm distance from a KRN sensor, a GC tube is fractured using static loading method as noted earlier. The outputs from the reference sensor under test and from V112 force transducer are recorded using a digital oscilloscope (Pico Scope 5242D, Pico Technology, St. Neots, UK). Recording normally used 14-bit resolution, 8 ns sampling interval, and 1 MΩ input impedance.

**Figure 7:**
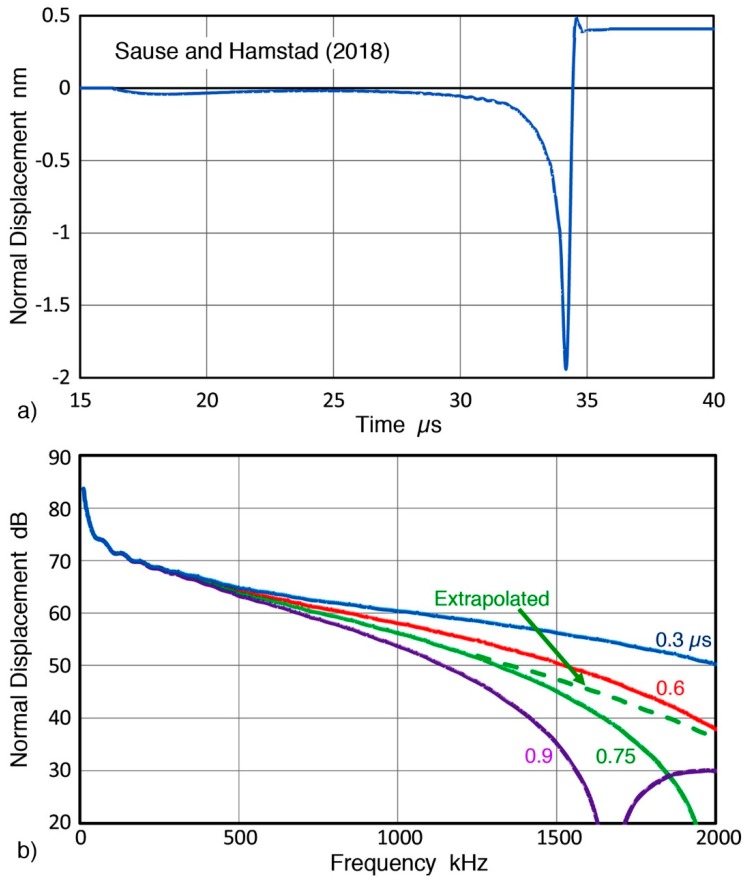
(**a**) Calculated and verified surface normal displacements from a GC fracture [23]. (**b**) FFT spectra of displacement function in Figure 5a with 0.3 µs rise time and its longer rise time modifications with 0.6, 0.75, and 0.9 µs. An extrapolated spectrum for 0.75 µs curve is given by green dash curve.

Calculated and verified surface normal displacements from a GC fracture are shown in Figure 7a. This displacement vs. time curve is from the FEM study of Sause and Hamstad [23] and for the case of aluminum medium at 100 mm distance from the GC tube fracture point. The cosine bell source function with the applied force of 10 N and the rise time of 0.3 µs were used, resulting in approximately 2 nm peak displacement. They calculated the displacements in the surface normal direction for steel, aluminum and a polymer at 100 mm distance from the point force, but only the steel results were published. This aluminum data was one of their unpublished results. Its power spectrum from FFT is plotted by a blue curve in Figure 7b. Also plotted in Figure 7b are modified versions using longer rise times of 0.6, 0.75, and 0.9 µs. This modification used the spectral differences of longer rise time cosine bell functions (Equation (1)). As was the case in Figure 5c, longer rise times produce steeper reduction with increasing frequencies above 0.5 MHz. This result points to a general trend, that is, the low frequency part of the displacement spectrum is less sensitive to the rise time increases. Thus, this part can scale the sensitivity levels when the rise time is uncertain since the applied force can be determined independently. Effects of windowing in FFT appear again for the rise times of 0.75 and 0.9 µs. An extrapolated correction is given as green dash curve for 0.75 µs rise time.

**Figure 8:**
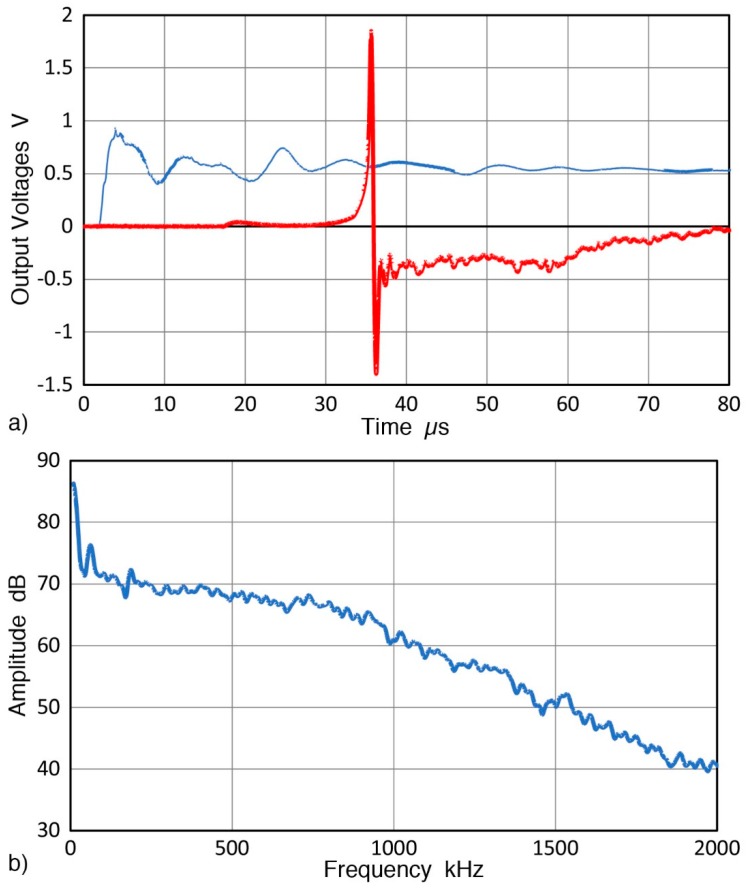
(**a**) Output voltages of reference sensor (KRNBB-PC) from GC fracture in red and force transducer (V112) in blue. Both were normalized to 10 N fracture force. (**b**) Averaged spectrum of eight received signals, also normalized to 10 N fracture force.

**Figure 9:**
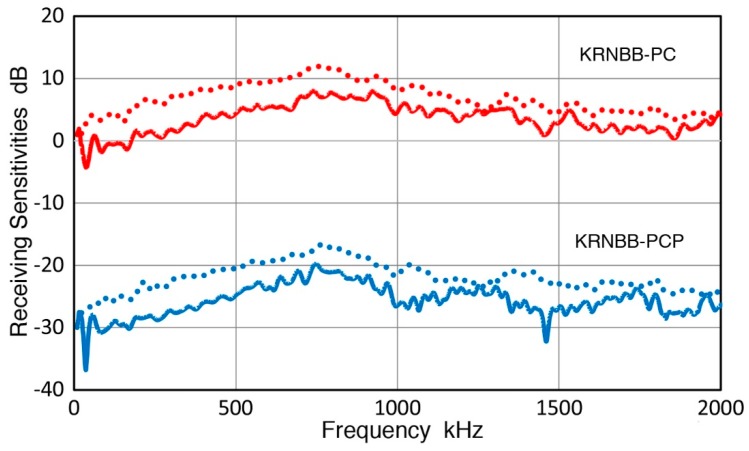
Rayleigh waves (RW) receiving sensitivities of the two KRN sensors in solid curves. Corresponding dotted curves show NW receiving sensitivities. KRNBB-PC in red and KRNBB-PCP in blue.

Two KRN sensors are characterized for their RW receiving sensitivities with GC fracture. The sensor was screwed into a C-shaped holder and pressed down with weights (see Figure 4). All the sensors and transducers used in this work are listed in Table 1. The GC fracture and sensor positions are in the middle along the diagonal direction of the aluminum transfer block. By placing thin sheets of sealing compound (2–3 mm thick) behind and along the sides surrounding the fracture and sensor positions, no apparent spurious reflections were observed for more than 150 µs. By 80 µs after GC fracture, the sensor output decayed to zero, as shown in Figure 8a. The amplitude of this signal was normalized to 10 N fracture force. This received signal waveform is similar to the calculated displacement, shown in Figure 7a. Its FFT spectrum was obtained and was combined with seven more from repeat tests, providing the averaged spectrum of the received signals, given in Figure 8b. Both Figure 7a and Figure 8a correspond to 10 N GC fracture force, and the displacement sensitivity of the tested sensor is found by spectral division. That is, the receiver spectrum in dB (Figure 8b) minus GC fracture spectrum in dB (Figure 7b using the extrapolated curve for 0.75 µs rise time). The RW receiving sensitivities of the two KRN sensors obtained are shown in Figure 9 as solid curves. The reference for the RW receiving sensitivity is 0 dB at 1 V/nm. Corresponding NW receiving sensitivity of the KRN sensors are shown by dotted curves of the same color (red for KRNBB-PC and blue for KRNBB-PCP). This figure clearly indicates the similarity of frequency dependence of RW and NW sensitivities, showing the highest peaks near 0.8 MHz. For both types, however, the NW receiving sensitivity is 3–6 dB higher than the RW receiving sensitivity. This higher NW sensitivity level was different from the conclusion of Sause and Hamstad [23], who found an identical sensitivity to all wave modes, longitudinal, Rayleigh, Lamb, and rod. The resolution of this discrepancy with their result requires direct laser interferometry, which will be conducted when it becomes accessible. The rest of this study assumes the validity of the approach used above.

Rayleigh wave (RW) calibration of two reference sensors, KRNBB types, was obtained using theoretical displacement calculation of GC fracture and detected signals from the reference sensors. The RW receiving sensitivity was similar to the corresponding NW receiving sensitivity, but its level was lower by 3 to 6 dB.

## 4. Rayleigh Wave Calibration of AE Sensors

The same procedure used for determining RW calibration of reference sensors can be applied to RW calibration of other sensors. However, the use of GC fracture can be avoided when another RW source is available. In this study, a 90° angle beam ultrasonic transducer is examined for the use as an alternate RW source. Here, an angle beam wedge is coupled to an ultrasonic transducer of 2.25 MHz center frequency (NDT Systems, Newport Beach, CA, USA, model C-16, 12.7 mm diameter). The wedge was made by Automation Industries (model 57K0403, Montrose, CO, USA), having 67° incident angle. A Vaseline couplant was used. The main advantages are the directivity of RW, the absence of wave intensities in the opposite or side directions, and straight wave front. The absence of longitudinal waves also contributed to the elimination of back and side reflections that necessitate the use of a large transfer block. It is also repeatable due to pulse excitation. At 100 mm from the front of the wedge, RW beam has intensity variation of ±0.15 dB over 12.7 mm width. The variation increased to ±0.7 dB for 19 mm width. The sensing element diameters of AE sensors, however, rarely exceed 12.7 mm and these are covered in the flat intensity zone of this RW source. Larger RW transducers are available if necessary.

**Figure 10:**
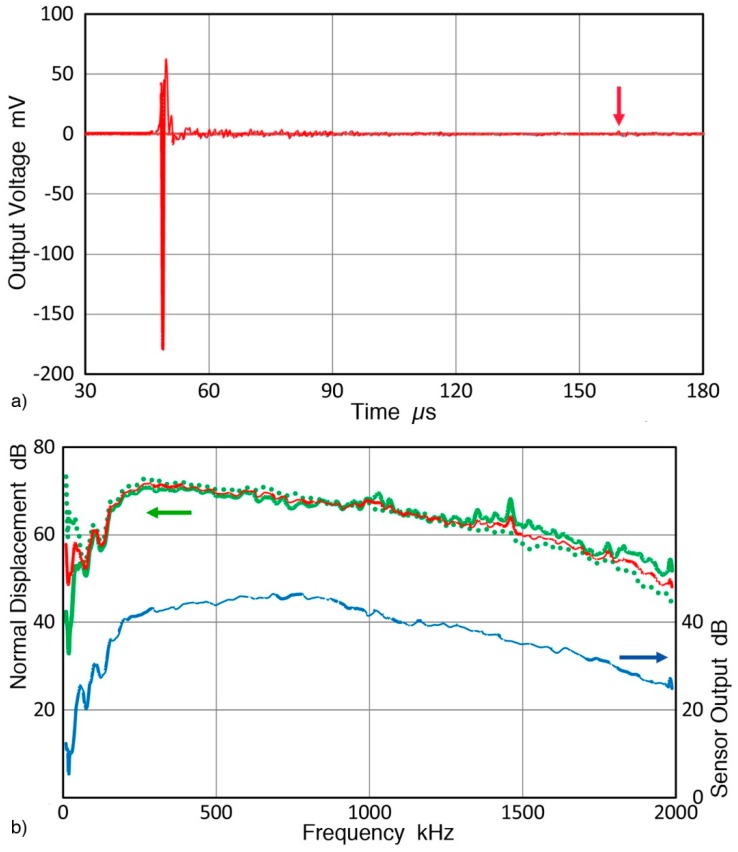
(**a**) RW pulses from angle beam ultrasonic transducer source received with a reference sensor, KRNBB-PCP. (**b**) The averaged spectrum of six received RW pulses (lower blue curve with right scale; 0 dB at 1 V). Also shown are the normal displacement spectrum of the RW pulses at the sensor position, (left scale; 0 dB at 1 nm). Green curve for KRNBB-PCP with waveform in Figure 10a, and green dot curve for KRNBB-PC. The averaged displacement spectrum is shown as red curve.

A received RW pulse signal from this angle beam ultrasonic transducer source with a reference sensor can be seen in Figure 10a. This example used KRNBB-PCP sensor and its sensitivity level is about 30 dB lower than KRNBB-PC version. In this set-up, with the wedge front at 77 mm from one corner of the block, only noticeable reflection occurred at 165 µs (red arrow) and its amplitude was down 40 dB from the peak signal. Less than ten more low amplitude reflections of similar or slightly higher levels were observed over a 10 ms period following the initial pulse. In most cases, signal up to 150 µs was subjected to spectral analysis using 8 ns sampling interval. With FFT, the intensity spectrum of the RW pulses was obtained. The averaged spectrum from six repeat tests (with six repeated mounting of the angle beam ultrasonic transducer and reference sensor) is shown in Figure 10b (blue curve with the right scale). By subtracting the RW receiving sensitivity of the corresponding reference sensor (Figure 9), the displacement spectrum of the RW pulses is determined. These results are also plotted in Figure 10b using the left scale. Green and red curves are for normal displacements at 100 mm distance from the angle beam transducer with the two reference sensors. That is, green curve is for KRNBB-PCP and green dot curve is for KRNBB-PC. The average of the two is given by red curve in Figure 10b (with the left scale). The displacement data showed large spreads below 50 kHz and this low frequency portion should be used with caution. At higher frequencies from 50 to 1300 kHz, the average difference between the two displacement spectra was 0.82 dB, which is a reasonably good agreement. This allows the use of the average curve with confidence. The difference was larger above 1300 kHz, possibly due to less consistency in sensor coupling to the transfer block.

For the calibration of an AE sensor, the sensor is placed by centering it to where the reference sensor was positioned using Vaseline couplant. The output of the AE sensor is recorded with a 10-kΩ termination at the oscilloscope input to simulate a typical AE preamplifier. Recording was taken to 180 µs from the input pulse, but a 150-µs portion was used for subsequent FFT. From this sensor output spectrum, the average displacement spectrum (red curve in Figure 10b) at the sensor position was subtracted, yielding the RW receiving sensitivity of the AE sensor tested.

**Figure 11:**
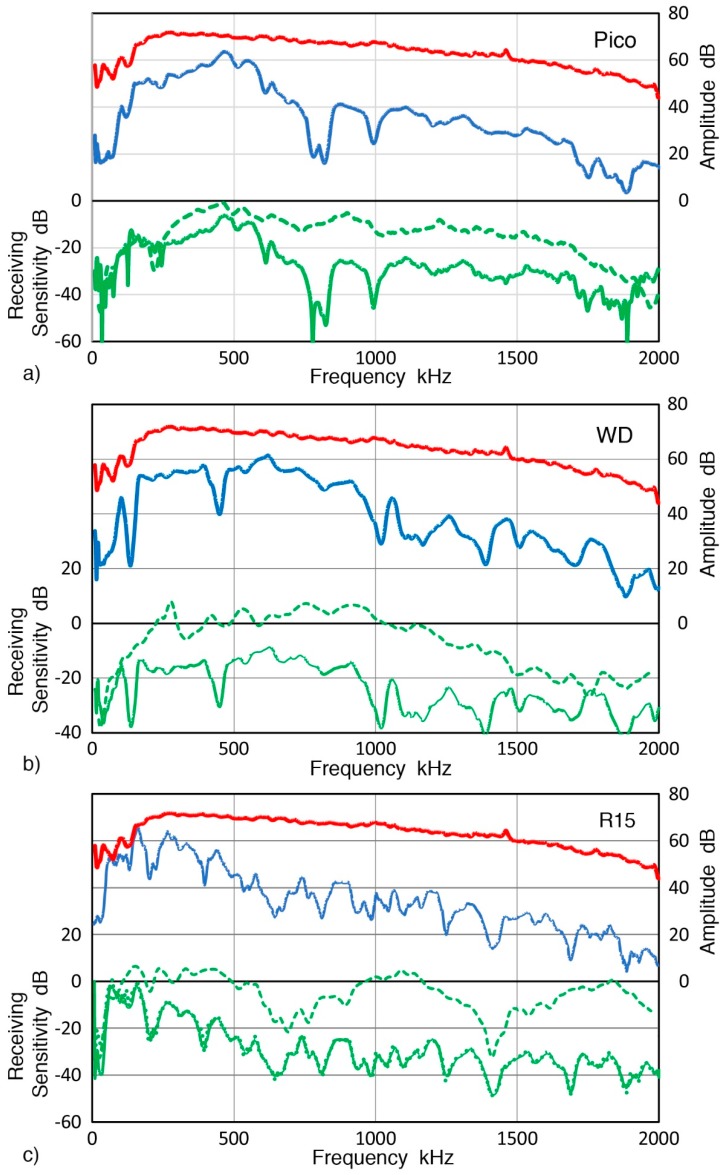
RW receiving sensitivity of three common AE sensors. Top red curve (right scale): averaged displacement spectrum from Figure 8b (0 dB in reference to 1 nm displacement); blue curve (right scale): sensor output spectrum in dB (0 dB in reference to 1 V sensor output); bottom green curve (left scale): RW receiving sensitivity with 0 dB in reference to 1 V/nm; green dash curve (left scale): NW receiving sensitivity for the same sensor. (**a**) Pico sensor, (**b**) WD sensor, and (**c**) R15 sensor. Green dot curve in Figure 11c gives RW receiving sensitivity using 5-ms long signal.

**Figure 12:**
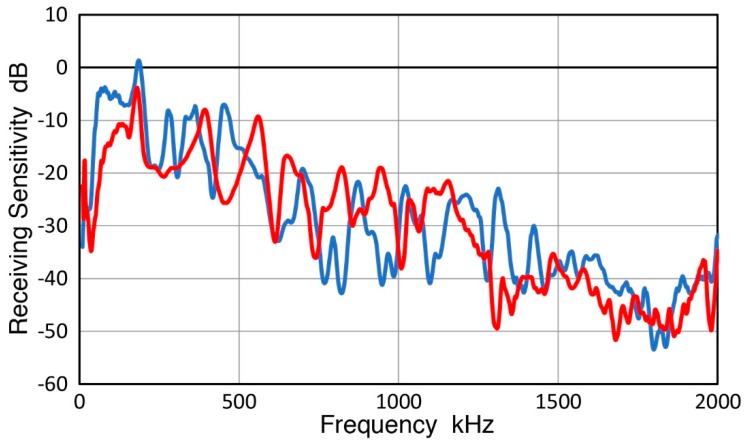
RW receiving sensitivity (0 dB in reference to 1 nm displacement) of two piezoelectric ceramic disks. Red curve: 12.7 mm diameter, 2 mm thick; blue curve: 11.4 mm diameter, 5.15 mm thick.

Results for three common AE sensors—Pico, WD, and R15—all from Physical Acoustics Corp. (Princeton Junction, NJ, USA), are shown in Figure 11. In these plots, the top red curve is for the displacement spectrum at the sensor position (0 dB in reference to 1 nm displacement) and the next blue curve shows sensor output spectrum in dB (0 dB in reference to 1 V sensor output; the right scale). The difference of the two produces the bottom green curve, representing the RW receiving sensitivity. This is given with the left scale with 0 dB in reference to 1 V/nm. Green dash curve above this curve is the corresponding receiving sensitivity to normally incident waves (NW) reported previously for the same sensor [32]. For all three AE sensors, NW receiving sensitivity was mostly higher than RW receiving sensitivity, typically by more than 20 dB. The differences increased with frequency as predicted from aperture effect calculations [16]. At low frequencies (<200 kHz for Pico, <100 kHz for WD, and <50 kHz for R15), however, the two receiving sensitivities were matching or close. RW receiving sensitivity spectra showed more dips from expected aperture effects. This will be discussed further in the next section. In case of R15 sensor, its output waveform was taken to 5 ms using longer sampling interval and the RW receiving sensitivity plotted in Figure 11c using green dots. This curve almost completely overlapped with that from the 150 µs output waveform. While reverberation from its resonant characteristics continued at low levels to 7 ms after the initial RW arrival, its receiving sensitivity remains unaffected. Similar features were found in two more resonant sensors, R6a and R15a and a flat response sensor, F30a (in Appendix A). This implies that signal duration of 150 µs used in the present study is adequate for the calibration of practical AE sensors. Figure 12 shows two RW receiving sensitivities for piezoceramic disks. One is nominally 1 MHz compression mode disk (PZT-5A, 12.7 mm diameter, 2.11 mm thick, Valpey Fisher Corp., Hopkinton, MA, USA) and the other is a sensing element from an AE sensor (unknown composition, 11.4 mm diameter, 5.15 mm thick, model AC175, AET Corp., Sacramento, CA, USA). Their RW sensitivities resembled that of R15 sensor in Figure 11c with many peaks and dips. For these three sensors, the highest sensitivity peak was from radial resonance at 156 to 185 kHz and most dips below 1 MHz roughly matched the frequencies expected from a sensing element of 12.7-mm diameter. Fifteen more sensors and transducers were evaluated and their RW, NW, and GW receiving sensitivities are given in the next section and in Appendix A.

The trend of different RW and NW sensitivities found here is consistent with the behavior observed by Matsuda et al. [34,35], using direct laser interferometry, as shown in Figure 2. However, it disagreed with the finding of Sause and Hamstad [23], who found that the receiving sensitivities to four wave types were identical. This appears to be from their use of the early parts of receiver responses, while received signal segments to 150 µs were used in this study. More discussion will follow later.

Notable features on RW receiving sensitivities observed in this study are (1) NW sensitivities are always higher than RW sensitivities, (2) two receiving sensitivities of small diameter KRN sensors are matched in shape, but RW being lower than NW, and (3) those of large diameter transducers show much lower RW receiving sensitivities than NW receiving sensitivities.

## 5. Discussion

### 5.1. Aperture Effects

When straight line Rayleigh waves are incident on a circular disk sensor, wave cancellation effects can be calculated and the resultant amplitude, U, is given by a Bessel function [16]:

U(a, f) = 2 J_1_(2πfa/V_R_)/(2πfa/V_R_), (2)

where a is the disk radius (mm), f is frequency (MHz), V_R_ is the Rayleigh wave velocity (mm/µs) and J_1_ is the Bessel function of the first kind, respectively. When V_R_ is taken to be 3.00 mm/µs (measured value for the aluminum block used was 2.91 mm/µs), the frequencies for amplitude minima, f_min_ (in kHz) are given by

f_min_ (x) = F_x_/a, (3)

with F_1_ = 1824, F_2_ = 3339, F_3_ = 4852, F_4_ = 6369, F_5_ = 7867, and F_6_ = 9367 (in kHz-mm). For common sensor element diameters of 3.2, 6.4 and 12.7 mm, the values of f_min_ under ~1.5 MHz are given in Table 2 below.

Data for Figure 11a shows f_min_ for a Pico sensor of 780, 824, 994 and 1888 kHz. If the last two are assumed to be the first and second minima, its sensing element is estimated as 3.67 mm diameter, which matches to its element diameter of 3.2 mm. For WD sensor (Figure 11b), frequencies of dips were 135, 452, 1026, and 1398 kHz. This sensor is known to have more than a single disk and 1026-kHz dip may be from a 3.6 mm diameter disk. Considering its 18-mm case diameter, lower dip frequencies are difficult to attribute to another disk element. It is possible that a cylindrical element is present as only two dips are found below 1 MHz. For R15 sensor (Figure 11c), known to be of a single disk design, frequencies of dips were (202, 395), 538, (668), 809, (931), 971, (1121), 1257, 1408, (1560), 1694, and (1886) kHz. While the first f_min_ was not observed, the second through 7th f_min_ predicted for 12.7 mm diameter had corresponding dips on the RW receiving sensitivity curve. Here, unmatched dips are indicated by parentheses. While the absence of the first order f_min_ was predicted in another calculation [39], it is not supported by the case of Pico sensor above. For the cases of bare piezoelectric disks (Figure 12), frequencies of dips were 265, (462, 612), 740, (856), 1009, and 1312 kHz for 12.7-mm PZT-5A disk (red curve). Good matching is found for the first, third to fifth f_min_ predicted (ignoring additional dips at 462, 612, and 856 kHz). For the second disk (11.4 mm), matching peaks were present for the first through the fifth-order f_min_ at 307, 624, 830, 1099, and 1392 kHz. The origins of extra dips are not explored, but these are expected from interactions of multiple resonances.

The aperture effects predicted by theory are followed in the case of straight line RW incident on a circular sensor. It was noticed previously that aperture effects were generally absent for bar and plate waves, reflecting many modes arriving at the sensor [28,29].

### 5.2. Sensitivities to other Guided Waves

**Figure 13:**
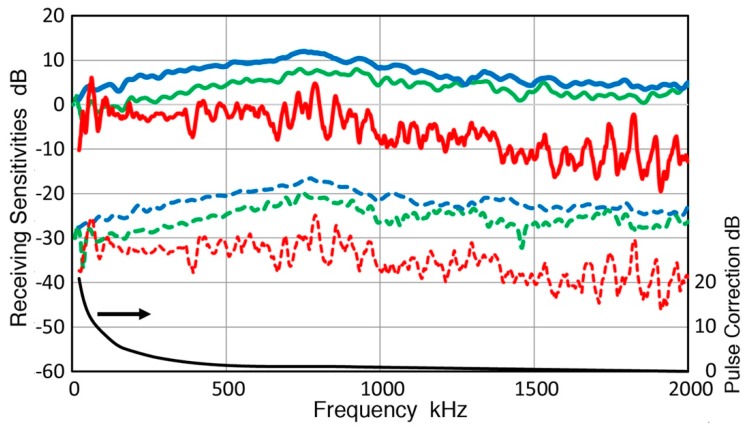
Receiving sensitivities of reference KRN sensors to three wave modes, NW (blue curves), RW (green curves) and other guided waves (GW, red curves). NW and RW sensitivities are from Figure 9. Previous GW receiving sensitivities [28,29] were corrected using spectral difference of two-pulse shapes, shown by black curve (use the right scale). Solid curves for KRNBB-PC and dash curves for KRNBB-PCP sensors. Use the left scale for all curves in color.

In addition to RW and NW, AE sensors need to respond to bar waves and Lamb (or plate) waves. Receiving sensitivities to bar waves (BW) and Lamb waves (LW), or collectively, guided waves (GW), were evaluated previously using displacement measurements conducted on a long aluminum bar of 3.6 m length [28,29]. Laser interferometry was used. A reference sensor (KRNBB-PCP as in this study) was calibrated and was used to determine the displacements on other bars and plates, excited by ultrasonic transducers attached on the end of a bar or a plate. GW receiving sensitivities were comparable to or higher than NW receiving sensitivities at low frequencies, but these always became lower than NW sensitivities above 0.5 MHz. Especially strange was a sharp rise in GW receiving sensitivities below 200 kHz. This behavior was reexamined in this study and was found to be due to an error in the choice of pulse input spectrum that excited BW in the original displacement calibration, which used a step pulse. When this work was resumed a few years later, a monopolar pulse spectrum was used erroneously. Thus, all GW calibration results in references [28,29] need to be corrected. Differences from the two-pulse FFT spectra were utilized and actual spectral correction is shown by black curve in Figure 13 (use the right scale).

Corrected GW receiving sensitivities of reference KRN sensors are shown with red curves in Figure 13. For both sensors, RW and GW sensitivities are comparable below 100 kHz, but GW values became lower at higher frequencies, the differences reaching 15 dB at 2 MHz. NW sensitivities were always higher than both RW and BW values (except for a GW peak at 50 kHz).

**Figure 14:**
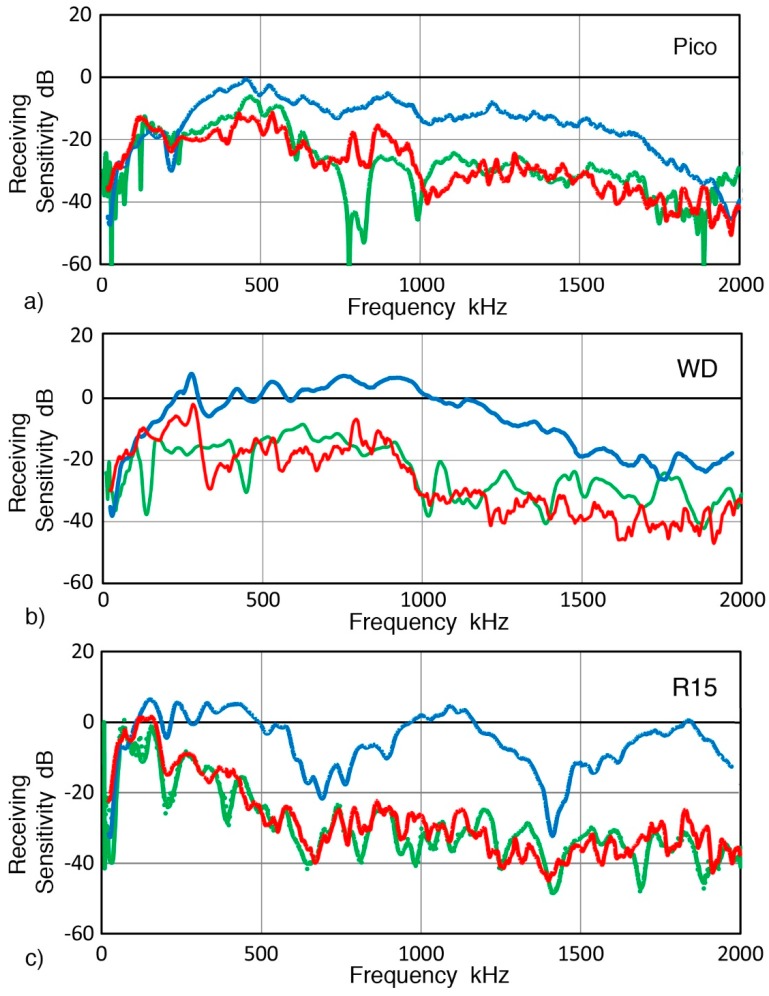
Receiving sensitivities of three AE sensors to three wave modes, NW (blue curves), RW (green curves), and GW (red curves). (**a**) Pico sensor, (**b**) WD sensor, and (**c**) R15 sensor.

For three AE sensors examined in Figure 11 (Pico, WD and R15, Physical Acoustics, Princeton Junction, NJ, USA), corrected GW curves generally matched RW curves as shown in Figure 14. Where peaks and dips were observed, however, differences were observed. This trend was evident at ~700–800 kHz for Pico, below 500 kHz for WD and at low frequency dips for R15 sensor. Again, both RW and GW sensitivities were persistently lower than NW sensitivities at f > 250–300 kHz. The three types of receiving sensitivities are compared for 15 more sensors and transducers and shown below.

**Figure 15:**
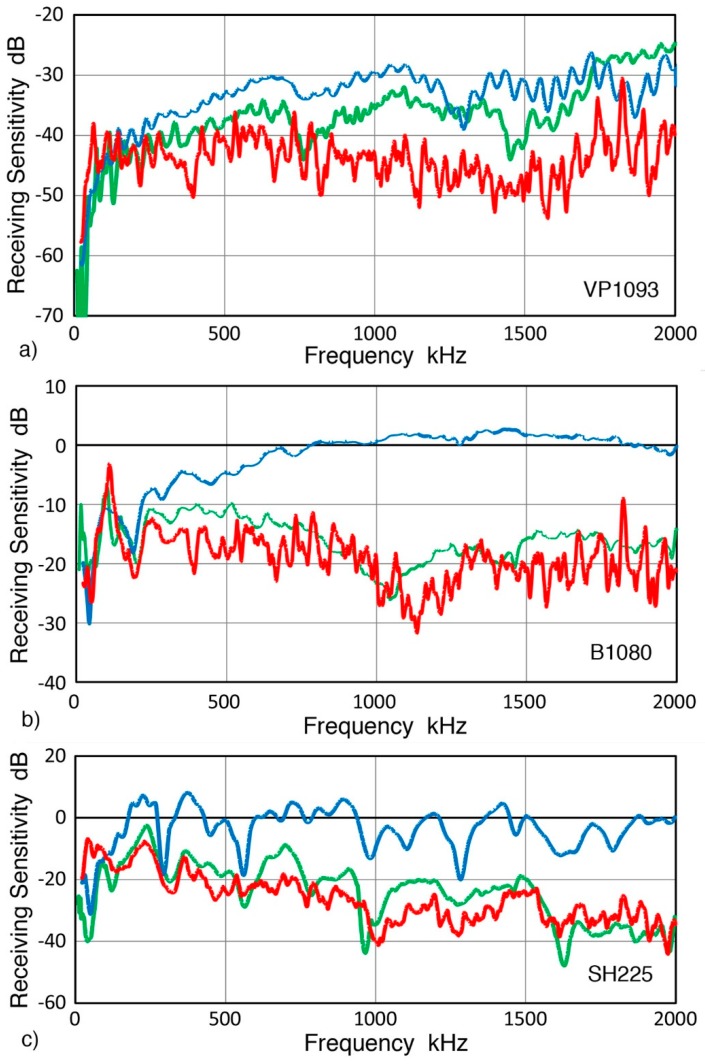
Receiving sensitivities of three AE sensors to three wave modes, NW (blue curves), RW (green curves), and GW (red curves). (**a**) VP-1093 sensor, (**b**) B1080 sensor, and (**c**) SH225 sensor.

Comparison of NW, RW and GW receiving sensitivities has shown that these always differ for a single sensor or transducer. All showed higher NW sensitivities. Only three 1-mm aperture sensors (two KRN sensors, shown in Figure 13, and VP-1093 from Valpey Fisher, shown in Figure 15a) exhibited GW sensitivities below RW values. Two more sensors, B1080 (Digital Wave Corp., Centennial, CO, USA) and SH225 (Dunegan Research Corp., San Juan Capistrano, CA, USA), also seemed to show this trend (Figure 15b,c), though these could be within a scatter band and additional tests are needed to reduce the scatter in the GW results. In contrast, 15 larger sensors and transducers showed higher NW receiving sensitivities and matched RW and GW sensitivities that are lower than NW values (see Figure 14 and in Appendix A). At low frequencies, the three sensitivities often merged, giving nearly identical sensitivities over a narrow range in some cases.

These differences in sensitivities are expected from wave overlapping on the sensing area and the number of wave propagation modes. For NW and RW, single mode is dominant at most frequencies, but several modes are expected in Lamb waves and numerous modes are present in bar waves. It is unclear how these modes interact and produce the frequency dependence observed. Since three sensitivities tend to merge at low frequencies even for large sensors (cf. Figure 14), this range may be evaluated further for clues. By examining the early parts of arriving waves, Sause and Hamstad [23] did show that four types of waves resulted in identical receiving sensitivity. More studies of small sensors appear to be useful for clarification.

### 5.3. Transfer Block Sizes

The use of a 90° angle beam ultrasonic transducer as the source of Rayleigh waves provides advantages. Because of the directivity, edge or side reflections are weak and these can be suppressed effectively by placing sheets of sealing compounds or modeling clay for attenuating RW. No longitudinal wave reflection was observed since longitudinal waves were refracted into Rayleigh waves. Three smaller transfer blocks were tried. These are aluminum blocks of 125 × 155 × 255 mm^3^, 50 × 125 × 305 mm^3^, and 63 × 101 × 285 mm^3^. In all cases, no spurious reflections interfered with RW measurements for at least 150 µs. This is comparable to the large standard block used in this work. When plates of 19 or 25 mm thickness were used for Lamb wave (LW) testing, however, resultant LW receiving sensitivities were close to RW sensitivities, but did show a few additional dips even though spurious reflections were not found even with increased oscilloscope sensitivities. It appears when the thickness is 100 mm or more, satisfactory calibration can be achieved. This corresponds to 77 kHz, above which frequency RW can be supported [57].

It should be noted that small transfer blocks can only be used with RW transducers. When NW transducers are excited, many reflections appear and these cannot be suppressed. Even using a meter-sized block, test duration is limited to about 100 µs [24].

Since the RW and GW sensitivities are similar for all practical AE sensors, one may substitute BW measurements for RW sensitivity calibration using a long, slender bar. This allows good transportability of a calibration bar even to field. A widely available longitudinal ultrasonic transducer can be dedicated for the BW calibration set-up. Only special item needed is a calibrated reference sensor. However, the scattering of GW sensitivities can be large depending on various conditions, sizes of bars and plates, symmetric or asymmetric excitation, types of transducer, etc. Most GW sensitivities used for comparison were averaged spectra of eight test conditions, and these showed scattering comparable to those in NW and RW sensitivities. When only two test conditions were used in the present work for VP1093, B1080, FC500, V104, V107, and V111, their GW sensitivity scattering doubled. Thus, a thicker block is required, but its width and length can be relatively limited as the above examples attest. Because of larger scattering of GW sensitivities and the similarities of the RW and GW sensitivities, the present study indicates that it is unnecessary to conduct separate GW calibration for most AE sensors.

## 6. Conclusions

Calibration of AE sensors and ultrasonic transducers for the detection of Rayleigh waves (RW) was conducted. Small aperture reference sensors were characterized first using the fracture of glass capillary tubes while getting accurate fracture force through the use of static fatigue behavior of glass. Another parameter for glass capillary (GC) fracture was its rise times, which were estimated using 10 MHz ultrasonic transducers as fast force gage. These were combined with a theoretical displacement calculation with FEM analysis, which was verified by laser interferometry [23]. These provided the foundation of RW calibration of the reference sensors. For the calibration of 18 commercial sensors and two piezoceramic disks, a 90° angle beam transducer was used to generate RW pulses on an aluminum transfer block. By determining the RW amplitude at a selected position, RW receiving sensitivities of a sensor under test was found by placing it at the designated sensor position and getting electrical output, which is known as the substitution method [31].

1. The fracture properties of commercially supplied GC tubes were evaluated and found to be similar to the GC tubes used elsewhere. However, the rise time of GC fracture in this study is estimated to be 0.62 to 0.75 µs, higher than the values of 0.2 to 0.3 µs reported in the earlier studies, reflecting the use of larger diameters of GC tubes.

2. Rayleigh wave calibration of two reference sensors, KRNBB types, was obtained using verified theoretical displacement calculation of GC fracture and estimated signal rise time from a force transducer. Accurate fracture force for each GC fracture was determined by the use of delayed fracture phenomenon of glass. The RW receiving sensitivities of the two reference sensors were similar to the corresponding normally incident wave (NW) receiving sensitivity in their frequency dependence, but were lower by 3 to 6 dB.

3. Notable features on RW receiving sensitivities of 20 sensors examined are (1) NW sensitivities are always higher than RW sensitivities, (2) differences between NW and RW receiving sensitivities are dependent on frequency and sensor size, (3) most sensors show comparable RW and GW receiving sensitivities, especially those of commonly used AE sensors, and (4) the receiving sensitivities of small aperture (1 mm diameter) sensors behave differently from larger sensors, with three types of the receiving sensitivities of a small aperture sensor being closer together in comparison to larger sensors.

4. Comprehensive examination of NW, RW and GW receiving sensitivities [26,27,28,29,30,32] suggests the following guidelines for practical AE sensor selection: (1) Some normal sized AE sensors exhibit good overall sensitivities. (2) Smaller AE sensors are better suited for RW and GW detection, but frequency-based selection can improve the detectability since each sensor has a range of frequency for best sensitivity. (3) Broadband ultrasonic transducers can be used for NW sensing, but are not suitable for use in RW and GW detection, especially above 0.5 MHz. (4) For effective AE detection, it is important to identify the signal propagation mode(s) and frequency content and to select appropriate AE sensors.

## Figures and Tables

**Table 1:** Transducers and sensors used.

Transducer Model	Manufacturer	Frequency MHz	Element Size mm
AE900M	Fuji Ceramics	1–4	3 ^E^
5045S	Fuji Ceramics	0.5–4	16 ^E^
KRNBB-PC ^A^	KRN Services	0.1–1	1
KRNBB-PCP	KRN Services	0.1–1	1
V111	Olympus	10	12.7
V112	Olympus	10	6.4
C-16	NDT Systems	2.25	12.7
Pico	Physical Acoustics	0.5	3.2
WD	Physical Acoustics	0.3–0.5	12.7 *
R15	Physical Acoustics	0.15	12.7
R6-alpha	Physical Acoustics	0.06	12.7 *
R15-alpha	Physical Acoustics	0.15	12.7 ^E^
F30-alpha	Physical Acoustics	0.2–0.7	12.7 *
VP1093	Valpey Fisher	0.1–10	1.0
V101	Olympus	0.5	25.4
V103	Olympus	1	12.7
V104	Olympus	2.25	25
V107	Olympus	5	25
FC500	AET Corp	2.25	19
µ30D	Physical Acoustics	0.3	8 ^E^
S9220	Physical Acoustics	0.9	8 ^E^
HD50	Physical Acoustics	0.5	3 ^E^
B1080 ^A^	Digital Wave	0.1–2	6.4 ^E^
SH-225	Dunegan Engineering	0.225	6.3 × 12.6 **

^A^ Amplifier included; ^E^ Estimated; * Multiple elements; ** Rectangular shear element.

**Table 2:** Values of the frequencies, F_x_, for amplitude minima, f_min_ (in kHz).

Diameter	F_1_	F_2_	F_3_	F_4_	F_5_	F_6_
3.2 mm	1140					
6.4 mm	570	1040	1510			
12.7 mm	288	526	764	1003	1239	1475

**Figure A1:**
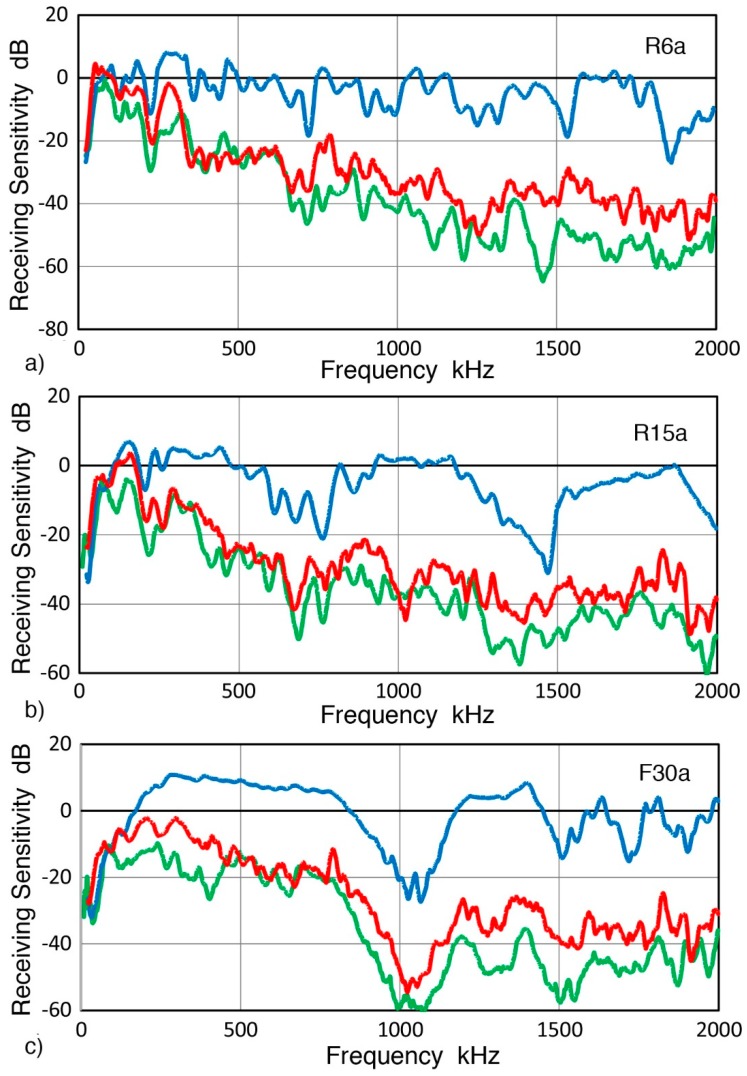
Receiving sensitivities of three AE sensors to three wave modes, NW (blue curves), RW (green curves), and GW (red curves). (**a**) R6a sensor, (**b**) R15a sensor, and (**c**) F30a sensor.

**Figure A2:**
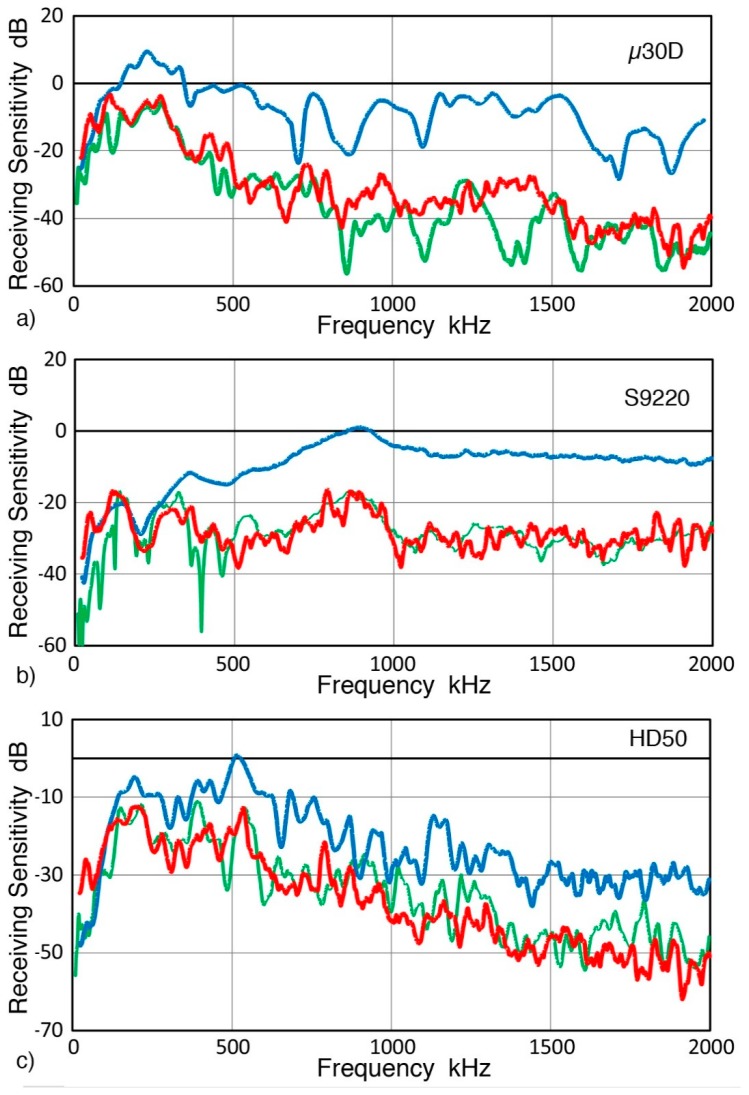
Receiving sensitivities of three AE sensors to three wave modes, NW (blue curves), RW (green curves), and GW (red curves). (**a**) µ30D sensor, (**b**) S9220 sensor, and (**c**) HD50 sensor.

**Figure A3:**
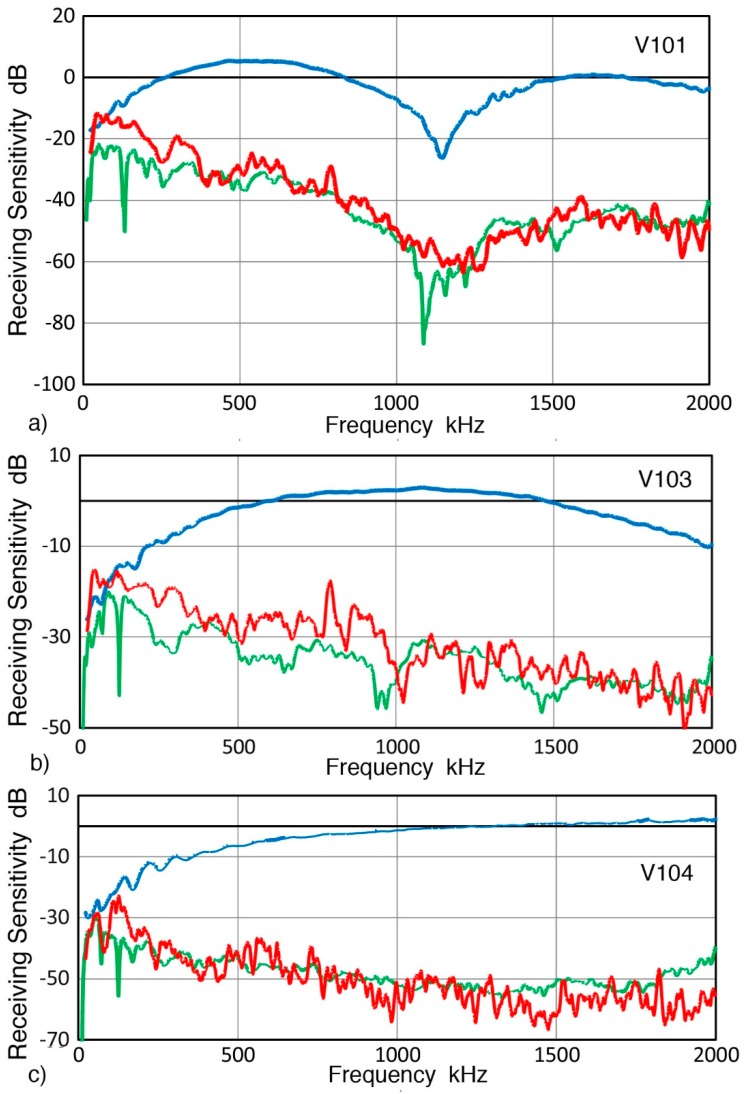
Receiving sensitivities of three ultrasonic transducers to three wave modes, NW (blue curves), RW (green curves), and GW (red curves). (**a**) Olympus V101 transducer, (**b**) V103 transducer, and (**c**) V104 transducer.

**Figure A4:**
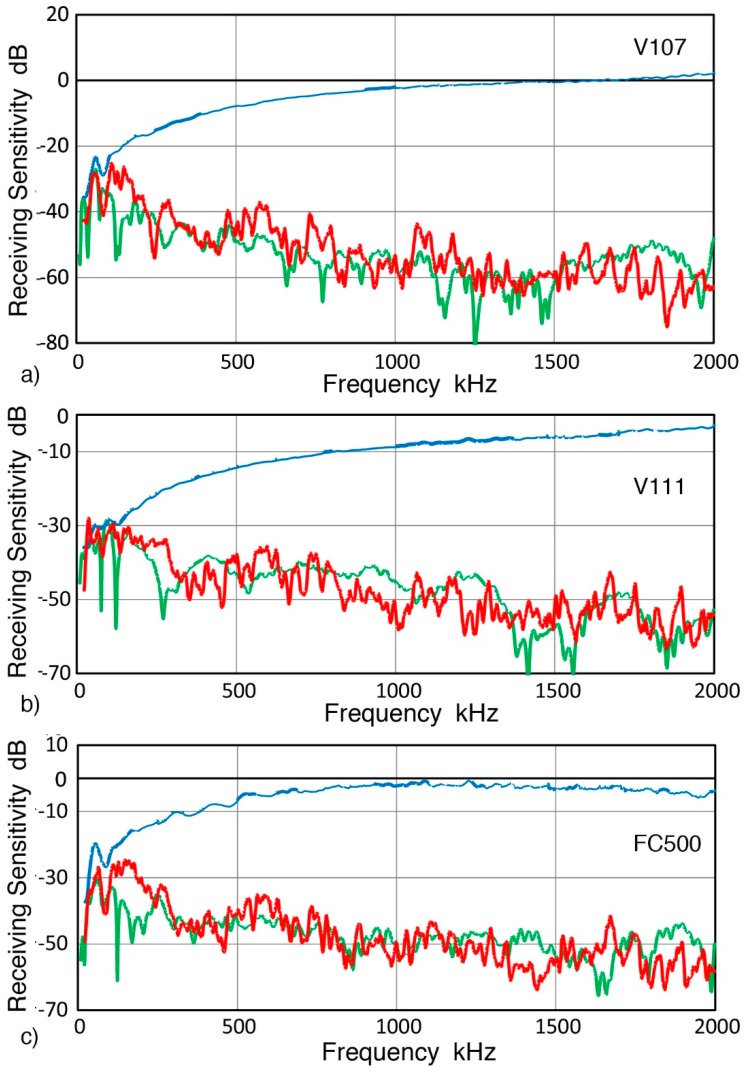
Receiving sensitivities of three ultrasonic transducers to three wave modes, NW (blue curves), RW (green curves), and GW (red curves). (**a**) Olympus V107 transducer, (**b**) V111 transducer, and (**c**) AET FC500 transducer.

## Appendix A. Receiving Sensitivities of Additional Sensors and Transducers

Plots of RW, NW, and GW receiving sensitivities for additional 12 sensors are presented here. Figure A1 shows receiving sensitivities of three AE sensors, R6a, R15a, and F30s (all from Physical Acoustics, Princeton Junction, NJ, USA), to three wave modes—NW (blue curves), RW (green curves), and GW (red curves). R15a sensor behavior is similar to its predecessor, R15 sensor (Figure 14c), but two others also showed comparable sensitivities. In all cases, RW and GW receiving sensitivities matched with GW values tending to be higher.

Figure A2 shows receiving sensitivities of three AE sensors, µ30D, S9220, and HD50 (all from Physical Acoustics), to three wave modes—NW (blue curves), RW (green curves), and GW (red curves). These three are smaller in size compared to the preceding three sensors and show less spread among the three receiving sensitivities and a good match between RW and GW sensitivities. These spectra were also closer to those of NW sensitivities than in Figure A1.

Figure A3 and Figure A4 show receiving sensitivities of six ultrasonic transducers to three wave modes—NW (blue curves), RW (green curves), and GW (red curves). These are (a) Olympus V101, (b) V103, and (c) V104 transducers in Figure A3 and (a) Olympus V107, (b) V111, and (c) FC500 (AET Corp.) transducers. In all six cases, a good match between RW and GW sensitivities was observed. However, the differences between NW and RW or BW sensitivities were larger than AE sensors. At 1 MHz, for example, the six AE sensors had differences of 10 to 18 dB (Figure A1 and Figure A2), while the second six ultrasonic transducers (in Figure A3 and Figure A4) showed 40–60 dB separation between NW and RW or GW sensitivities. A large difference was found even for the same element size of 12.7 mm between R15a and V103. All the ultrasonic transducers were of broadband type with heavy damping, whereas AE sensors are designed for high sensitivity with minimal damping. The role of damping on NW detection is for the suppression of back reflection, but the primary vibration can act on the sensing piezoelectric element during the first passage. On the other hand, the sensing element responds to the net displacement on its entire face in detecting RW or GW. When the waves pass across the sensor face, parts of them are attenuated by the damping materials behind the sensing element since the RW or GW wavelength is more than the thickness of sensing elements (0.2–4 mm). RW and GW also propagate at the couplant layer, which adds to attenuation for larger transducers. These are likely to contribute substantially to the observed sensitivity reduction.

From the data of the additional AE sensors and ultrasonic transducers, the following recommendations emerge. (1) Ultrasonic transducers examined here are not suitable for use in RW and GW detection, especially above 0.5 MHz. (2) Smaller AE sensors are better suited for RW and GW detection, but frequency-based selection can improve the detectability since each sensor has a range of frequency for best sensitivity, e.g., 225 kHz for SH225, 500 kHz for Pico, and HD50, 900 kHz for S9220. (3) Some normal sized AE sensors exhibit good RW and GW sensitivities, e.g., WD, R6a, R15a, and F30a. Thus, for RW and GW detection, it is especially important to follow a long-standing recommendation of “know the signal frequencies.”
